# Investigation of the Dominant Microbiota in Ready-to-Eat Grasshoppers and Mealworms and Quantification of Carbapenem Resistance Genes by qPCR

**DOI:** 10.3389/fmicb.2018.03036

**Published:** 2018-12-17

**Authors:** Vesna Milanović, Andrea Osimani, Andrea Roncolini, Cristiana Garofalo, Lucia Aquilanti, Marina Pasquini, Stefano Tavoletti, Carla Vignaroli, Laura Canonico, Maurizio Ciani, Francesca Clementi

**Affiliations:** ^1^Dipartimento di Scienze Agrarie, Alimentari ed Ambientali, Università Politecnica delle Marche, Ancona, Italy; ^2^Dipartimento di Scienze della Vita e dell'Ambiente, Università Politecnica delle Marche, Ancona, Italy

**Keywords:** edible insects, antibiotic resistance, PCR-DGGE, carbapenemase genes, qPCR

## Abstract

In this study, 30 samples of processed edible mealworms (*Tenebrio molitor* L.) and 30 samples of grasshoppers (*Locusta migratoria migratorioides*) were obtained from producers located in Europe (Belgium and the Netherlands) and Asia (Thailand) and subjected to PCR-DGGE analyses. The PCR-DGGE analyses showed that species in the genus *Staphylococcus* were predominant in the samples of mealworms from Belgium and grasshoppers from the Netherlands; species in the genus *Bacillus* were detected in the samples of mealworms and grasshoppers from Thailand. Moreover, *Weissella cibaria/confusa*/spp. was found in grasshoppers from Belgium. Since data concerning the role of novel foods such as edible insects in the dissemination of carbapenem resistance are currently lacking, the quantification of five carbapenemase encoding genes (*bla*_NDM−1_, *bla*_VIM_, *bla*_GES_, *bla*_OXA−48_, and *bla*_KPC_) by qPCR was also carried out in all the samples under study. The genes coding for GES and KPC were not detected in the analyzed samples. A very low frequency of *bla*_OXA−48_ (3%) and *bla*_NDM−1_ (10%) genes was detected among mealworms. In contrast, grasshoppers were characterized by a high incidence of the genes for OXA-48 and NDM-1, accounting for 57 and 27% of the overall grasshopper samples, respectively. The *bla*_VIM_ gene was detected exclusively in two grasshopper samples from Thailand, showing only 7% positivity. The analysis of variance showed that all the effects (producers, species, and producers × species) were statistically significant for *bla*_NDM−1_, whereas for *bla*_OXA−48_ and *bla*_VIM_, no significant effects were detected for the same source of variation. Further studies are necessary to assess the possible role of edible insects as reservoirs for the resistance to carbapenems and to understand the correlation with the insect microbiota. Furthermore, an intensified surveillance plan examining the occurrence of carbapenemase encoding genes in the food chain and in environmental compartments is needed for a proper risk assessment. In such a context, the appropriate use of antimicrobials represents the main preventive action that should always be applied.

## Introduction

The use of insects for human consumption is a frequent practice worldwide, mainly in Thailand and other Asian countries, Africa, America, and Australia (van Huis et al., 2013; Schlüter et al., 2017). In Europe, edible insects represent an innovative and uncommon protein source, although in some European countries, especially in the Netherlands and Belgium, the rearing and the industrial production of edible insects is gradually increasing (ANSES, 2014; Schlüter et al., 2017). Indeed, several promising aspects are associated with insect consumption since insects (i) are generally characterized by a positive nutrient profile in terms of high-quality proteins and amino acids, good lipids, vitamins, minerals, and fiber, (ii) are easy to breed, and (iii) cause lower emissions of greenhouse gases and ammonia than traditional livestock (Klunder et al., 2012; van Huis et al., 2013; Schlüter et al., 2017). Due to these numerous nutritional, social, and environmental benefits, edible insects are considered the “food of the future” and categorized as novel foods by Regulation (EU) No 2015/2283 of the European Parliament and of the Council.

Among the edible insects, mealworms and grasshoppers are included within those that are already commercialized as food in EU countries (Schlüter et al., 2017) and that have been partially investigated for the presence of relevant pathogens or potentially pathogenic microorganisms (Ali et al., 2010; Klunder et al., 2012; Stoops et al., 2016; Garofalo et al., 2017; Osimani et al., 2017a, 2018a) as well as for the presence of transferable resistances to antibiotics (Milanović et al., 2016; Osimani et al., 2017b,c, 2018b).

Carbapenems are broad-spectrum β-lactam antibiotics, currently considered the last-line antibiotics for the treatment of severe human infections caused by multidrug-resistant Gram-negative bacteria (EFSA BIOHAZ Panel, 2013; Guerra et al., 2014; Woodford et al., 2014). The production of carbapenemases, which are β-lactamases capable of hydrolyzing carbapenems and almost all β-lactams, represents the main mechanism of resistance to carbapenems (Tzouvelekis et al., 2012; Doi and Paterson, 2015; Fischer et al., 2017). Among the carbapenemases, the plasmid-acquired class A serine-β-lactamases KPC (*Klebsiella pneumoniae* carbapenemase, 17 variants) and GES (Guiana extended spectrum, 9 variants), class B metallo-β-lactamases VIM (Verona integron-encoded metallo-beta-lactamase, 40 variants) and NDM (New Delhi metallo-beta-lactamase, 10 variants), and class D serine-β-lactamases including OXA carbapenemases (Carbapenem-hydrolyzing oxacillinase) such as OXA-48 are among the most common and important from an epidemiological point of view and are thus the main clinical concern (Queenan and Bush, 2007; Grundmann et al., 2010; Miriagou et al., 2010; Walsh, 2010; Cantón et al., 2012; Monteiro et al., 2012; Pfeifer et al., 2012; Guerra et al., 2014; Woodford et al., 2014; Fischer et al., 2017). Carbapenemases are encoded by genes that are easily transferable among bacteria by horizontal gene transfer events since they are located on mobile genetic elements, thus increasing their worldwide spread among bacteria in different reservoirs (Tzouvelekis et al., 2012; Woodford et al., 2014; Doi and Paterson, 2015; Fischer et al., 2017). Indeed, during the last few years, the spread of carbapenem resistance has increased, especially in the Enterobacteriaceae, as well as in non-fermenters such as *Pseudomonas* spp. and *Acinetobacter* spp. and in non-pathogenic bacteria such as *Stenotrophomonas* spp. and *Myroides* spp. (Miriagou et al., 2003; Grundmann et al., 2010; Nordmann et al., 2011, 2012; Cantón et al., 2012; Tzouvelekis et al., 2012; Guerra et al., 2014; Doi and Paterson, 2015; Morrison and Rubin, 2015). In the last decade, the rapid and global dissemination of infections caused by carbapenemase-producing Enterobacteriaceae (CPE) in hospitals and healthcare institutions is of great concern since these outbreaks are often associated with high mortality rates due to the limited and inadequate alternative treatments (Nordmann et al., 2011; Doi and Paterson, 2015; Rossolini, 2015; Grundmann et al., 2017; Zhang et al., 2017). From all of these data, the current opinion is that the acquired carbapenemases are a primary pressing public health threat related to antibiotic resistance (AR) (Tzouvelekis et al., 2012; EFSA BIOHAZ Panel, 2013; Woodford et al., 2014; Doi and Paterson, 2015).

Although the occurrence of carbapenemases was first discovered and mainly investigated at hospitals and healthcare facilities, scientific studies reporting carbapenemase-producers, and carbapenemase-encoding genes (CEG) in animals, the environment and food are increasingly frequent. Specifically, carbapenem resistance has been detected in livestock and in their environments in France, Germany, Switzerland, the USA, and China (Guerra et al., 2014; Webb et al., 2016; Zurfluh et al., 2016; Fischer et al., 2017); companion animals and wildlife (Guerra et al., 2014; Woodford et al., 2014); aquatic environments (Zurfluh et al., 2013; Guerra et al., 2014; Woodford et al., 2014; Fernando et al., 2016); retail chicken meat from Egypt (Abdallah et al., 2015); vegetables and seafood from Asia, India and Brazil (Guerra et al., 2014; Morrison and Rubin, 2015; Zurfluh et al., 2015, 2016). This suggests that non-human sources may be reservoirs of CPE and CEG. Since it is widely recognized that the food chain is one of the main routes for the introduction of antibiotic-resistant bacteria and their genes into the human digestive tract, and for the diffusion and spread of AR in human pathogens (Clementi and Aquilanti, 2011; Rolain, 2013; Milanović et al., 2017), there is a need for intensified surveillance of the occurrence of CEG in the food chain and in different environmental compartments. This is also underscored by the European Food Safety Authority, which has recently recognized the need to improve European legislation to ensure the monitoring of carbapenem resistance in animals and food (EFSA BIOHAZ Panel, 2013).

Based on all these premises, edible insects deserve great attention in terms of safety, including the assessment of the microbiota and of the incidence of AR genes, in particular CEG.

Therefore, in order to obtain an overview of the predominant bacterial species in samples of processed edible mealworms (*Tenebrio molitor* L.) and grasshoppers (*Locusta migratoria migratorioides*) obtained from producers in Europe (Belgium and the Netherlands) and Asia (Thailand), the total microbial DNA was analyzed by culture-independent PCR-DGGE.

Concerning the AR issue, it is worth noting that currently, only a few scientific studies are available on the occurrence of transferable AR genes in edible insects (Milanović et al., 2016; Osimani et al., 2017b,c, 2018b; Vandeweyer et al., 2018), and to the authors' knowledge, none have been published on the detection of CEG.

To address this gap, the edible insect samples under study were subjected to screening by quantitative PCR (qPCR) of five among the most common carbapenem resistance genes (*bla*_NDM−1_, *bla*_VIM_, *bla*_GES_, *bla*_OXA−48_, and *bla*_KPC_) (Monteiro et al., 2012). Statistical analyses were performed to determine if edible insect species (mealworms and grasshoppers) or geographical location correlated with the occurrence of carbapenem resistance genes in this study.

## Materials and Methods

### Sampling

Thirty samples of edible mealworms and 30 samples of grasshoppers (boiled, dried, and salted) were purchased via the internet from dealers located in Europe (Belgium and the Netherlands) and Asia (Thailand). Ten mealworm and ten grasshopper samples from each country were collected and marked as follows: TN1-TN10 (mealworms from the Netherlands, Producer 1), TB1-TB10 (mealworms from Belgium, Producer 2), TT1-TT10 (mealworms from Thailand, Producer 3), GN1-GN10 (grasshoppers from the Netherlands, Producer 1), GB1-GB10 (grasshoppers from Belgium, Producer 2), and GT1-GT10 (grasshoppers from Thailand, Producer 3). All the samples were provided in sealed plastic containers and delivered at ambient temperature via international shipping. No information was available on the rearing and hygiene conditions of processing, transport and storage applied to these edible insects before marketing.

### Bacterial DNA Extraction

Total microbial DNA was extracted directly from the insect samples using PowerFood Microbial DNA Isolation Kit (Mo Bio Laboratories, Carlsbad, CA, USA) as described by Osimani et al. (2017a). The extracted DNA was quantified and checked for the purity using a NanoDrop ND 1000 (Thermo Fisher Scientific, Wilmington, DE, USA) and then standardized to 2 ng μL^−1^ for qPCR assays and to 25 ng μL^−1^ for PCR-DGGE analysis. The effective extraction of bacterial DNA was confirmed by conventional PCR amplification of 2 μL (50 ng) of extracted DNA suspensions in a My Cycler Thermal Cycler (BioRad Laboratories, Hercules, CA, USA) using universal prokaryotic primers 27F and 1495R as described by Osimani et al. (2015).

### PCR-DGGE Analysis

The equal portions of DNAs extracted from insects were mixed together with the goal of obtaining six pooled samples (TB, TN, TT, GB, GN, and GT), each representing an insect type (mealworms and grasshoppers) and country of origin (Belgium, the Netherlands, and Thailand). The amplification products obtained from the 27F-1495R primer pair as described above were purified using the Illustra GFX PCR DNA and Gel Band Purification Kit (GE Healthcare Life Sciences, Buckinghamshire, UK). Subsequently, 2 μL of the purified PCR products was reamplified using the universal prokaryotic primers U968GC (added with GC clamp) and L1401 (Muyzer et al., 1993; Randazzo et al., 2002) following the PCR conditions previously described by Aquilanti et al. (2013). Following amplification, 5 μL of the PCR reaction was loaded on a 1.5% agarose gel together with a 100 bp molecular weight marker (HyperLadder™ 100 bp) to check for the expected product size of 480 bp. Twenty microliters of these PCR amplicons were analyzed by DGGE (30–60% urea-formamide denaturing gradient; 4 h at 130 V) using DCode Universal Mutation Detection System (Bio-Rad Laboratories) as described by Garofalo et al. (2015). All DGGE bands visible under UV light were excised from the gel, and the DNA was eluted overnight at 4°C in 50 μL of molecular biology grade water (Garofalo et al., 2008) and reamplified via PCR as described above, but with the forward primer U968 lacking the GC clamp. The PCR products were sent to Genewiz (Takeley, UK) for purification and sequencing, and the obtained sequences were identified at species level as described above by Osimani et al. (2018c).

### Reference Strains

DNA extracted from five reference strains (Table 1), each carrying one of the carbapenem resistance genes under study, was used as positive control in the qPCR reactions and for the construction of qPCR standard curves.

**Table 1:** Bacterial reference strains carrying carbapenems resistance genes, used as positive controls in the qPCR reactions.

**Bacterial strain**	**Carbapenems resistance gene**
BAA 2146_ *Klebsiella pneumoniae*	*bla*_NDM−1_
LEMC_VIM-1_ *Pseudomonas aeruginosa*	*bla*_VIM_
LEMC_GES-1_ *Pseudomonas aeruginosa*	*bla*_GES_
LEMC_OXA-48_ *Klebsiella pneumoniae*	*bla*_OXA−48_
ATCC 1705_ *Klebsiella pneumoniae*	*bla*_KPC_

### qPCR

Absolute quantification of each carbapenemase gene (*bla*_NDM−1_, *bla*_VIM_, *bla*_GES_, *bla*_OXA−48_, and *bla*_KPC_) in the insect samples was performed by qPCR in a Mastercycler® ep realplex machine (Eppendorf, Hamburg, Germany) using the qPCR primers and cycling conditions described by Monteiro et al. (2012). To check for product specificity, all cycles were followed by a melt curve step analysis with temperature gradually increasing from 65 to 95°C by 0.2°C/s. Each qPCR reaction consisted of 4 μL (8 ng) of the extracted DNA; 5 μL of Type-it 2X HRM PCR Master Mix (Qiagen, Hilden, Germany) containing HotStarTaq Plus DNA Polymerase, EvaGreen Dye, an optimized concentration of Q-solution, dNTPs and MgCl_2_; 0.2 μM of forward and reverse primers for each gene; and nuclease-free molecular biology grade water to a final reaction volume of 10 μL. The exogenous standards for each gene were prepared by qPCR amplification of the DNA extracted from the reference strains (Table 1) as described above but in a final reaction volume of 25 μL. The correct melting temperatures (Tm) and sizes of the obtained PCR products were checked by melting curve analysis and electrophoresis on a 1.5% agarose gel, respectively. The Illustra GFX PCR DNA and Gel Band Purification Kit (GE Healthcare Life Sciences) was used for the purification of the amplicons following the manufacturer's instructions. The quantity and the purity of the products were determined using the NanoDrop ND 1000 (Thermo Fisher Scientific). The calculation of each gene copy number was performed using an online calculator (www.idtdna.com) based on the mass and size of the purified qPCR products. The standard curves were created by qPCR amplification of 10-fold serial dilutions of exogenous standards. The amplification efficiencies were estimated from the slopes of the standard curves, and the correlation coefficients (*R*^2^) (Stolovitzky and Cecchi, 1996) were calculated automatically by Mastercycler® ep realplex software. To determine the qPCR detection limit for each gene, the standard curves were generated in the range from ~1 to 10^7^ gene copies per reaction.

For the absolute quantification of CEG, the DNAs extracted from mealworms and grasshoppers were run along with the 10-fold serial dilutions of the standards prepared as described above. The gene copy number of each gene detected in the analyzed insect samples was determined using the slope of the corresponding standard curves. The baseline and threshold calculations were performed automatically by the Mastercycler® ep realplex software. In addition to melting curve analysis, the correct sizes of the amplification products were checked by electrophoresis on 1.5% agarose gels using 100 bp DNA Ladder (HyperLadder™ 100 bp, Bioline, UK) as a molecular weight marker. Moreover, the accuracy of the amplification reactions was validated by the sequencing (Genewiz) of randomly selected positive samples (TB6, GT7, and GN1 for the *bla*_OXA−48_ gene; GT2 and TT8 for the *bla*_NDM−1_ gene; GT7 and GT8 for the *bla*_VIM_ gene). The resulting sequences were compared with those from the GenBank database (http://www.ncbi.nlm.nih.gov) using Basic Local Alignment Search Tool (BLAST) (Altschul et al., 1990). All qPCR reactions were performed in triplicate and the results were expressed as the mean gene copy number per ng of DNA ± standard deviation for each gene.

### Statistical Analysis

Descriptive statistics, calculated on 20 samples for each producer and on 30 samples for each insect species were carried out for the *bla*_OXA−48_, *bla*_NDM−1_, and *bla*_VIM_ gene copies by computing the means ± standard deviation.

After first checking for conformance to a normal distribution and identification of outliers, analysis of variance (ANOVA) was carried out using JMP statistical software version 11.0.0 (SAS Institute Inc., Cary, NC, USA) to test the following main effects: producers (Belgium, Thailand, the Netherlands), insect species (mealworm, grasshoppers) and producer × species.

Principal component analysis (PCA) was applied to discriminate among mealworms and grasshoppers coming from different producers (Belgium, the Netherlands, and Thailand), and the presence of genes related to resistance to carbapenems (*bla*_NDM−1_, *bla*_VIM_, *bla*_GES_, *bla*_OXA−48_, and *bla*_KPC_). PCA was carried out using the Unscrambler 7.5 software (CAMO ASA, Oslo, Norway). The mean data were normalized to neutralize any influence of hidden factors. PCA provides a graphical representation of the overall differences in terms of distribution of genes between the insects coming from different producers.

## Results and Discussion

In the present study, the microbiota of commercialized read-to-eat grasshoppers and mealworms from different countries was investigated via PCR-DGGE, as well as the quantification and distribution of five common CEG within the same matrices, in order to have a more complete picture of some safety aspects related to edible insects.

### Determination of Microbial Diversity

To have an overview of the predominant bacterial species found in the edible insects considered in this study, the total microbial DNA was extracted from the samples, the DNAs were mixed in order to obtain six pooled samples, each representing an insect type (mealworms, grasshoppers) and country of origin (Belgium, the Netherlands, Thailand) and then analyzed by a culture-independent PCR-DGGE method. The results obtained are reported in Table 2.

**Table 2:** Sequencing results of the bands excised from the DGGE gel obtained from the amplified fragments of pooled bacterial DNA extracted directly from mealworms and grasshoppers.

**Sample**	**Closest relative**	**% Identity^a^**	**Acc.no^b^**
TB	*Staphylococcus warneri*	98	MH211317
	*Staphylococcus pasteuri*	98	MH158278
	*Staphylococcus* sp.	98	MH191108
	*Staphylococcus kloosii*	99	CP027846
	*Staphylococcus cohnii*	99	KY012323
	*Staphylococcus* sp.	99	KY865752
TN	*Exiguobacterium* sp.	82	MG859628
	*Eikenella corrodens*	90	KU663108
	*Eikenella* sp.	90	KU738863
	*Neisseria shayeganii*	90	KM462144
TT	*Bacillus* sp.	99	MG757948
	*Bacillus* sp.	99	LT899995
GB	*Weissella cibaria*	99	CP027427
	*Weissella confusa*	99	MF327674
	*Weissella* sp.	99	MG814036
GN	*Staphylococcus haemolyticus*	99	MH179468
	*Staphylococcus argenteus*	99	LC378381
	*Staphylococcus* sp.	99	MH021651
	*Staphylococcus hominis*	99	MF327701
	*Staphylococcus aureus*	99	MG976640
GT	*Bacillus sonorensis*	99	KY243955
	*Bacillus subtilis*	99	KU172428
	*Bacillus amyloliquefaciens*	99	KJ126909
	*Bacillus axarquiensis*	99	KJ126897
	*Bacillus* sp.	100	LT899995

*TB- pool of 10 (TB1-TB10) mealworm samples from Belgium; TN- pool of 10 (TN1-TN10) mealworm samples from the Netherlands; TT- pool of 10 (TT1-TT10) mealworm samples from Thailand; GB- pool of 10 (GB1-GB10) grasshopper samples from Belgium; GN- pool of 10 (GN1-GN10) grasshopper samples from the Netherlands; GT- pool of 10 (GT1-GT10) grasshopper samples from Thailand*.

a *Percentage of identical nucleotides in the sequence obtained from the DGGE bands and the sequence of the closest relative found in the GenBank database*.

b *Accession number of the sequence of the closest relative found by a BLAST search*.

The dominant species found in mealworms from Belgium and grasshoppers from the Netherlands belonged to the genus *Staphylococcus*, and species in the genus *Bacillus* were found in mealworms and grasshoppers from Thailand. Grasshoppers from Belgium were positive for *Weissella cibaria*/*confusa*/spp., while bacterial species with a percentage identity below 97% were found in mealworms from the Netherlands. It is interesting to note that the predominance of lactic acid bacteria (LAB), and in particular the *Weissella* spp., has been reported for processed and fresh grasshoppers from Belgium and the Netherlands (Stoops et al., 2016; Garofalo et al., 2017; Osimani et al., 2017a), thus suggesting that the specific rearing conditions may have selected for this microbial group or that this bacterial species is intrinsically associated with this edible insect.

The genera *Staphylococcus* and *Bacillus* identified among the other pooled samples of mealworms and grasshoppers may contain pathogenic species such as *Staphylococcus aureus* and *Bacillus cereus*, and these data are in agreement with other studies on the microbiota in fresh and processed mealworms and grasshoppers (Stoops et al., 2016; Garofalo et al., 2017). The presence of *Staphylococcus* members may be due to an environmental contamination occurring during human handling or processing. Indeed, these insects were boiled and salted, and since *Staphylococcus* spp. is a halophile bacterium usually predominating in environments with low microbial competition, it could have found conditions suitable for growth.

The lack of detection of bacterial DNA belonging to the Enterobacteriaceae has been already reported by Osimani et al. (2018c), although it is generally reported that Enterobacteriaceae represents a predominant bacterial group in the edible insect gut microbiota (Stoops et al., 2016; Garofalo et al., 2017; Osimani et al., 2017b). These data suggest that good manufacturing practices were applied during rearing and processing of the insects and/or that a degutting step may have been applied. A further explanation is that members of the Enterobacteriaceae family could be present within the processed insect samples but with a lower abundance in respect with other microbial groups. This latter hypotesis is supported by the fact that these microorganisms had previously found below the detection limit of microbial counts (<1 Log cfu g^−1^) in the same samples (Osimani et al., 2017b,c).

Additionally, the lack of detection of bacterial DNA belonging to Pseudomonadaceae is unusual, but it is possible, as suggested for Enterobacteriaceae, that PCR-DGGE was not able to detect members of this microbial group if they were in the minority relative to the others.

### Quantification and Distribution of carbapenem Resistance Genes

This study represents the first report on the screening of five carbapenemase encoding genes (*bla*_NDM−1_, *bla*_VIM_, *bla*_GES_, *bla*_OXA−48_, and *bla*_KPC_) in processed edible mealworms (*T. molitor* L.) and edible grasshoppers (*L. migratoria migratorioides*) from producers located in Europe (Belgium and the Netherlands) and Asia (Thailand). The identification of the genes coding for these carbapenemases in the samples of edible insects under investigation was conducted by using qPCR. As previously underlined by Monteiro et al. (2012), molecular assays are considered the best solutions for the rapid detection of carbapenem resistance genes and for the identification of the resistance mechanism. The detection limit, defined as the lowest concentration at which 95% of the positive samples are detected was <10 gene copies per reaction for all the genes. The efficiencies of the qPCR reactions were 1.00 for the genes *bla*_OXA−48_, *bla*_VIM_, and *bla*_GES_; 1.01 for the gene *bla*_KPC_ and 0.96 for the gene *bla*_NDM−1_. The *R*^2^ was 1.000 for the genes *bla*_OXA−48_ and *bla*_NDM−1_; 0.999 for the gene *bla*_GES_, 0.996 for the gene *bla*_KPC_ and 0.995 for the gene *bla*_VIM_. Moreover, the specificity of the primers used for the amplification of carbapenem resistance genes was confirmed by the results of the sequencing of randomly selected positive samples, which showed >97% similarity with the corresponding gene sequences deposited in the GenBank database. In more detail, the results of the BLAST analysis for the *bla*_OXA−48_ gene showed 98% of the similarity with the sequences deposited in GenBank such as *K. pneumoniae* (CP031374), *Citrobacter freundii* (MG430338), *Enterobacter ludwigii* (MG436907), *Pantoea agglomerans* (MG436898), *Escherichia coli* (NG_055499), *Shewanella* sp. (NG_055475), and *Proteus mirabilis* (KT175900); for the *bla*_NDM−1_ gene 99% similarity with the *Enterobacter hormaechei* (AP018835), *K. pneumoniae* (AP018834), *Raoultella planticola* (MH257689), *E. coli* (CP021206), *Pseudomonas aeruginosa* (KT364224), and *Acinetobacter baumannii* (KU180703); and for the *bla*_VIM_ gene 98% similarity with the *K. pneumoniae* (MH071811), *C. freundii* (NG_061412), *Paenibacillus* sp. (KR822172), *E. coli* (MF169879), *E. hormaechei* (LT991955), *Kluyvera cryocrescens* (MG228427), *Alcaligenes faecalis* (KY623659), *Klebsiella oxytoca* (NG_050362), and *Enterobacter cloacae* (CP030081).

The results of the qPCR quantification of carbapenem resistance genes in samples of ready-to-eat edible mealworms and grasshoppers produced in the Netherlands, Belgium and Thailand are reported in Tables 3, 4.

**Table 3:** Results of qPCR quantification of carbapenemase genes in samples of ready-to-eat edible mealworms produced in the Netherlands (TN1-TN10), Belgium (TB1-TB10) and Thailand (TT1-TT10).

**Producer**	**Samples**	**Carbapenemase resistant genes (gene copies ng^−1^± standard deviation)**				
		***bla***_**GES**_	***bla***_**KPC**_	***bla***_**OXA-48**_	***bla***_**NDM-1**_	***bla***_**VIM**_
1	TN1	n.d.	n.d.	n.d.	n.d.	n.d.
	TN2	n.d.	n.d.	n.d.	2.29 ± 0.31	n.d.
	TN3	n.d.	n.d.	n.d.	n.d.	n.d.
	TN4	n.d.	n.d.	n.d.	n.d.	n.d.
	TN5	n.d.	n.d.	n.d.	n.d.	n.d.
	TN6	n.d.	n.d.	n.d.	n.d.	n.d.
	TN7	n.d.	n.d.	n.d.	n.d.	n.d.
	TN8	n.d.	n.d.	n.d.	0.94 ± 0.13	n.d.
	TN9	n.d.	n.d.	n.d.	n.d.	n.d.
	TN10	n.d.	n.d.	n.d.	n.d.	n.d.
	TN % of positivity for each determinant	n.dr.	n.dr.	n.dr.	20.0	n.dr.
2	TB1	n.d.	n.d.	n.d.	n.d.	n.d.
	TB2	n.d.	n.d.	n.d.	n.d.	n.d.
	TB3	n.d.	n.d.	n.d.	n.d.	n.d.
	TB4	n.d.	n.d.	n.d.	n.d.	n.d.
	TB5	n.d.	n.d.	n.d.	n.d.	n.d.
	TB6	n.d.	n.d.	1.81 ± 0.01	n.d.	n.d.
	TB7	n.d.	n.d.	n.d.	n.d.	n.d.
	TB8	n.d.	n.d.	n.d.	n.d.	n.d.
	TB9	n.d.	n.d.	n.d.	n.d.	n.d.
	TB10	n.d.	n.d.	n.d.	n.d.	n.d.
	TB % of positivity for each determinant	n.dr.	n.dr.	10.0	n.dr.	n.dr.
3	TT1	n.d.	n.d.	n.d.	n.d.	n.d.
	TT2	n.d.	n.d.	n.d.	n.d.	n.d.
	TT3	n.d.	n.d.	n.d.	n.d.	n.d.
	TT4	n.d.	n.d.	n.d.	n.d.	n.d.
	TT5	n.d.	n.d.	n.d.	n.d.	n.d.
	TT6	n.d.	n.d.	n.d.	n.d.	n.d.
	TT7	n.d.	n.d.	n.d.	n.d.	n.d.
	TT8	n.d.	n.d.	n.d.	3.38 ± 0.18	n.d.
	TT9	n.d.	n.d.	n.d.	n.d.	n.d.
	TT10	n.d.	n.d.	n.d.	n.d.	n.d.
	TT % of positivity for each determinant	n.dr.	n.dr.	n.dr.	10.0	n.dr.
	Overall % of positivity for each determinant	n.dr.	n.dr.	3.0	10.0	n.dr.

*n.d., not detected. n.dr., not determined*.

**Table 4:** Results of qPCR quantification of carbapenemase genes in samples of ready-to-eat edible grasshoppers produced in the Netherlands (GN1-GN10), Belgium (GB1-GB10) and Thailand (GT1-GT10).

**Producer**	**Samples**	**Carbapenemase resistant genes (gene copies ng^−1^± standard deviation)**				
		***bla***_**GES**_	***bla***_**KPC**_	***bla***_**OXA-48**_	***bla***_**NDM-1**_	***bla***_**VIM**_
1	GN1	n.d.	n.d.	1.17 ± 0.19	n.d.	n.d.
	GN2	n.d.	n.d.	0.24 ± 0.02	n.d.	n.d.
	GN3	n.d.	n.d.	n.d.	n.d.	n.d.
	GN4	n.d.	n.d.	0.86 ± 0.09	n.d.	n.d.
	GN5	n.d.	n.d.	n.d.	n.d.	n.d.
	GN6	n.d.	n.d.	n.d.	n.d.	n.d.
	GN7	n.d.	n.d.	0.89 ± 0.01	n.d.	n.d.
	GN8	n.d.	n.d.	0.30 ± 0.04	n.d.	n.d.
	GN9	n.d.	n.d.	n.d.	2.64 ± 0.19	n.d.
	GN10	n.d.	n.d.	n.d.	n.d.	n.d.
	GN % of positivity for each determinant	n.dr.	n.dr.	50.0	10.0	n.dr.
2	GB1	n.d.	n.d.	0.57 ± 0.02	n.d.	n.d.
	GB2	n.d.	n.d.	0.73 ± 0.01	1.85 ± 0.05	n.d.
	GB3	n.d.	n.d.	0.30 ± 0.08	2.25 ± 0.12	n.d.
	GB4	n.d.	n.d.	0.83 ± 0.01	n.d.	n.d.
	GB5	n.d.	n.d.	0.35 ± 0.11	n.d.	n.d.
	GB6	n.d.	n.d.	0.24 ± 0.01	n.d.	n.d.
	GB7	n.d.	n.d.	n.d.	n.d.	n.d.
	GB8	n.d.	n.d.	n.d.	n.d.	n.d.
	GB9	n.d.	n.d.	0.58 ± 0.03	n.d.	n.d.
	GB10	n.d.	n.d.	0.25 ± 0.05	6.37 ± 0.22	n.d.
	GB % of positivity for each determinant	n.dr.	n.dr.	80.0	30.0	n.dr.
3	GT1	n.d.	n.d.	0.30 ± 0.1	10.31 ± 0.52	n.d.
	GT2	n.d.	n.d.	8.65 ± 1.29	70.38 ± 3.01	n.d.
	GT3	n.d.	n.d.	n.d.	n.d.	n.d.
	GT4	n.d.	n.d.	n.d.	24.88 ± 0.71	n.d.
	GT5	n.d.	n.d.	n.d.	n.d.	n.d.
	GT6	n.d.	n.d.	n.d.	n.d.	n.d.
	GT7	n.d.	n.d.	16.56 ± 0.48	51.13 ± 1.59	392.25 ± 1.77
	GT8	n.d.	n.d.	n.d.	n.d.	42.83 ± 6.82
	GT9	n.d.	n.d.	0.59 ± 0.01	n.d.	n.d.
	GT10	n.d.	n.d.	n.d.	n.d.	n.d.
	GT % of positivity for each determinant	n.dr.	n.dr.	40.0	40.0	20.0
	Overall % of positivity for each determinant	n.dr.	n.dr.	57.0	27.0	7.0

*n.d., not detected. n.dr., not determined*.

Regarding mealworms, none of the samples were positive for the genes *bla*_GES_, *bla*_KPC_, and *bla*_VIM_, while only sample TB6 was found positive for *bla*_OXA−48_ (3% positivity) and samples TN2, TN8, and TT8 were positive for *bla*_NDM−1_ (10% positivity) (Table 3).

Regarding grasshoppers, the genes *bla*_GES_ and *bla*_KPC_ were not detected in any of the analyzed samples while only two samples from Thailand (GT7 and GT8) were positive for the *bla*_VIM_ gene (7% positivity). Interestingly, a high prevalence of *bla*_OXA−48_ was noted (57% positivity), followed by *bla*_NDM−1_ (27% positivity) (Table 4). Specifically, *bla*_OXA−48_ was prevalent in 80% of the samples from Belgium, in 50% of the samples from the Netherlands and in 40% of the samples from Thailand. The highest frequency of *bla*_NDM−1_ was found among samples from Thailand (40%), followed by samples from Belgium (30%) and the Netherlands (10%) (Table 4).

All the insect samples analyzed in this study were previously screened for the presence of 12 selected genes coding for resistance to tetracyclines [*tet*(M), *tet*(O), *tet*(S), and *tet*(K)], macrolide-lincosamide-streptogramin B (MLS_B_) [*erm*(A), *erm*(B), *erm*(C)], vancomycin (*vanA* and *vanB*), beta-lactams (*blaZ* and *mecA*) and aminoglycosides [*aac(6*′*)-Ie aph(2*″*)-Ia* referred as *aac-aph*] through PCR and nested PCR assays (Osimani et al., 2017b,c). It is interesting to note that the mealworms samples TN2 and TN8 from the Netherlands were also found to be positive for the presence of genes coding for resistance to tetracyclines [*tet*(M), *tet*(K), *tet*(S)] and erythromycin [*erm*(B), *erm*(C)], while sample TB6 was positive for genes coding for resistance to tetracyclines [*tet*(M), *tet*(K)], and sample TT8 from Thailand was positive for genes coding for resistance to tetracyclines [*tet*(K)], erythromycin [*erm*(B)], and aminoglycosides (*aac-aph*) (Osimani et al., 2017b). Moreover, among grasshoppers, almost all of the samples that were found to be positive for *bla*_OXA−48_, *bla*_NDM−1_, and *bla*_VIM_ previously showed positivity for AR genes coding for resistance to tetracyclines [*tet*(M), *tet*(S), *tet*(K)], erythromycin [*erm*(B), *erm*(C)], aminoglycosides (*aac-aph*) and beta-lactams (*blaZ*) (Osimani et al., 2017c).

The average levels of gene copies ng^−1^ in the 60 samples of edible insects were as follows: 0.59 ± 2.39 with a minimum value of 0 and a maximum value of 16.56 for *bla*_OXA−48_, 2.94 ± 11.53 with a minimum value of 0 and a maximum value of 70.37 for *bla*_NDM−1_, 7.25 ± 50.85 with a minimum value of 0 and a maximum value of 392.25 for *bla*_VIM_.

Descriptive statistics on 20 samples from each producer are shown in Table 5. In addition, descriptive statistics on 30 samples from each insect species are reported in Table 6.

**Table 5:** Descriptive statistics on 20 samples for each producer.

	**Belgium**			**Thailand**			**The Netherlands**		
	***bla***_**OXA-48**_	***bla***_**NDM-1**_	***bla***_**VIM**_	***bla***_**OXA-48**_	***bla***_**NDM-1**_	***bla***_**VIM**_	***bla***_**OXA-48**_	***bla***_**NDM-1**_	***bla***_**VIM**_
Mean	0.28	0.52	0.00	1.31	8.00	21.75	0.17	0.29	0.00
*SD*	0.45	1.51	0.00	4.07	19.22	87.73	0.36	0.77	0.00
Minimum	0.00	0.00	0.00	0.00	0.00	0.00	0.00	0.00	0.00
Maximum	1.81	6.37	0.00	16.56	70.37	392.25	1.17	2.64	0.00

*SD, standard deviation. Values are expressed as gene copies ng^−1^*.

**Table 6:** Descriptive statistics on 30 samples for each insect species.

	**Mealworm**			**Grasshoppers**		
	***bla***_**OXA-48**_	***bla***_**NDM-1**_	***bla***_**VIM**_	***bla***_**OXA-48**_	***bla***_**NDM-1**_	***bla***_**VIM**_
Mean	0.06	0.22	0	1.11	5.66	14.5
*SD*	0.33	0.74	0	3.31	15.96	71.77
Minimum	0	0.00	0	0	0	0
Maximum	1.81	3.37	0	16.56	70.37	392.25

*SD, standard deviation. Values are expressed as gene copies ng^−1^*.

The analysis of variance (Table 7) showed that all the variables (producers, species, and producers × species) had significant effects (*P* < 0.05) on the frequency of *bla*_NDM−1_, whereas for *bla*_OXA−48_ and *bla*_VIM_, no significant effects were detected for the same source of variation.

**Table 7:** ANOVA results for *bla*_OXA−48_, *bla*_NDM−1_, *bla*_VIM_.

**Source of variation**	**df**	***bla***_****OXA-48****_			***bla***_****NDM-1****_			***bla***_****VIM****_		
		**SS**	**MS**	***P***	**SS**	**MS**	***P***	**SS**	**MS**	***P***
Producer	2	15.60	7.80	0.24	769.50	384.75	0.04	6309.68	3154.84	0.30
Species	1	16.64	16.64	0.08	443.85	443.85	0.05	3154.84	3154.84	0.27
Producer × species	2	18.26	9.31	0.19	736.79	368.39	0.04	6309.68	3154.84	0.30

*Df, degrees of freedom. Significant at P < 0.05. SS, sum of squares. MS, mean square. P-value*.

Regarding the distribution of *bla*_OXA−48_ and *bla*_VIM_, multiple comparisons between ACC Least Square Means (LSM) carried out using the Tukey test showed no significant differences among samples from different producers or insect species. Regarding *bla*_NDM−1_, multiple comparisons (Tukey HSD) showed that insect species, but not the origin of the sample, had a significant correlation (*P* < 0.05) with the frequency of the gene.

PCA did not discriminate between the presence of genes encoding resistance to carbapenems among mealworms and grasshoppers coming from different producers. In contrast, in the previous studies on the occurrence of transferable ARs in ready-to-eat edible insects, PCA showed a differentiation among producers, thus suggesting that different rearing and clinical practices associated with different countries may have played a role in the variability observed (Milanović et al., 2016; Osimani et al., 2017b,c).

As reported by Schlüter et al. (2017), it is presumed that the rearing and processing conditions applied to edible insects will comply with the same food safety regulations as for livestock farming. The use of carbapenems is prohibited in food-producing animals in all countries (OIE, 2015; Webb et al., 2016). Notwithstanding, scientific studies reporting CPE and CEG in livestock and their environment are progressively more frequent (Guerra et al., 2014; Webb et al., 2016; Zurfluh et al., 2016; Fischer et al., 2017). Furthermore, in the last EFSA report on antimicrobial resistance in zoonotic and indicator bacteria from humans, animals and food in 2015, the presumptive extended-spectrum beta-lactamase (ESBL)-/AmpC-/carbapenemase-production in *Salmonella* and *E. coli* was monitored in humans, meat (pork and beef), fattening pigs and calves for the first time (EFSA and ECDC, 2017). Varying occurrence/prevalence rates of ESBL-/AmpC-producers were observed between countries, and carbapenemase-producing *E. coli* were detected in single samples of pig meat and from fattening pigs from two Member States (EFSA and ECDC, 2017). These data indicate that other antimicrobial classes could indirectly select CPE outside the hospital setting and that the rapid dissemination of CPE is also promoted by CEG located on plasmids transmissible by horizontal gene transfer events (Tzouvelekis et al., 2012; Woodford et al., 2014). As reviewed by Caniça et al. (2015), AR comprises a dynamic network that involves several environmental niches (e.g., water, soil, and plants) and different reservoirs (e.g., husbandry, hospitals, wild animal, human settings, human hand, food and global trade in foodstuffs) in which the path of dissemination and dynamics of AR genes has to be taken into consideration in order to understand and prevent the AR transmission and spread. Therefore, it is possible to hypothesize that, irrespective of the use of carbapenems in the edible insect rearing, the CEG may derive from the substrates used for feed or from surfaces and hands of operators or from treatments applied for processing, in addition to transport and storage. It is also interesting to note that grasshoppers and mealworms have different dietary habits since grasshoppers are grass-feeders whereas mealworms are usually reared on cereal-based matrices; therefore, the differences in terms of presence and distribution of CEG among these insect species may derive from different rearing practices and substrates.

## Conclusion

Edible insects such as grasshoppers and mealworms represent a novel food that deserves attention in terms of safety, including the assessment of the incidence of AR genes. The investigation of the microbiota of the mealworm and grasshopper samples in this study revealed the presence of potential pathogenic and non-pathogenic species.

Scientific studies reporting carbapenemase-producing microorganisms and CEG in animals, the environment and food are increasingly frequent. The data presented in this study is the first attempt aimed at determining the incidence of CEG among samples of commercialized ready-to-eat grasshoppers and mealworms from Belgium, the Netherlands and Thailand. Although further studies are necessary to understand the correlation of CEG with the insect microbiota and to assess the possible role of edible insects as reservoirs of resistance to carbapenems, an intensified surveillance plan examining the occurrence of CEG in the food chain and in different environmental compartments, along with a prudent use of carbapenems and antimicrobials in general, are primary measures that should be applied.

## Author Contributions

VM and AR carried out molecular analyses. MP, ST, LC, and MC performed statistical analyses. CG wrote the manuscript. AO, LA, CV, and FC critically analyzed the results and revised the final manuscript.

### Conflict of Interest Statement

The authors declare that the research was conducted in the absence of any commercial or financial relationships that could be construed as a potential conflict of interest.
